# Heterozygous *Med13l* mice recapitulate a developmental growth delay and craniofacial anomalies seen in *MED13L* syndrome

**DOI:** 10.1002/dvdy.70079

**Published:** 2025-09-08

**Authors:** Anna K. Leinheiser, Timothy T. Nguyen, Kayla M. Henry, Mariela Rosales, Eric Van Otterloo, Chad E. Grueter

**Affiliations:** ^1^ Department of Internal Medicine, Division of Cardiovascular Medicine, Francois M. Abboud Cardiovascular Research Center, Fraternal Order of Eagles Diabetes Research Center University of Iowa Iowa City Iowa USA; ^2^ Interdisciplinary Graduate Program in Genetics University of Iowa Iowa City Iowa USA; ^3^ Iowa Institute for Oral Health Research, Department of Periodontics, College of Dentistry and Dental Clinics, Department of Anatomy and Cell Biology, Carver College of Medicine University of Iowa Iowa City Iowa USA; ^4^ Craniofacial Anomalies Research Center University of Iowa Iowa City Iowa USA

**Keywords:** craniofacial anomalies, developmental growth delay, haploinsufficiency, *MED13L* syndrome, mediator, mouse model, neural crest

## Abstract

**Background:**

Gene transcription is crucial for embryo and postnatal development and is regulated by the Mediator complex. Mediator is comprised of four submodules, including the kinase submodule (CKM). The CKM consists of MED13, MED12, CDK8, and CCNC. In mammals, there are paralogs for CKM components, including MED13L, MED12L, and CDK19. Neurological disorders have been associated with mutations in CKM genes including *MED13L* syndrome. *MED13L* syndrome is generally characterized as a haploinsufficiency of MED13L with a broad phenotypic response due in part to a wide range of de novo mutations.

**Results:**

We developed a *Med13l* heterozygous (HET) mouse model with an exon 11 deletion to evaluate whether *Med13l* HET mice are a viable research tool to study human phenotypes. We characterized our mouse model using growth, cardiovascular, and skeletal readouts. We observed *Med13l* HET mice are smaller than wildtype (WT) littermates, and over 60% of them exhibited one of two craniofacial anomalies: a pug snout with midface hypoplasia or a crooked snout. We also observed discontinuous squamosal sutures in a subset of our *Med13l* HETs.

**Conclusions:**

*Med13l* HET mice recapitulate *MED13L* syndrome phenotypes including a developmental growth delay and craniofacial anomalies. *Med13l* HET mice represent a novel research tool for *MED13L* syndrome.

## Key Findings

- We developed a Med13L mouse model that partially recapitulates aspects of the human *MED13L* syndrome. *Med13L* homozygous deletion of exon 11 results in embryonic lethality. *Med13L* heterozygous mice develop midfacial anomalies. *Med13L* heterozygous mice exhibit developmental growth delay.

## 1 INTRODUCTION

Precise spatial and temporal regulation of genes is paramount for normal embryological and postnatal development.^1^, ^2^, ^3^, ^4^, ^5^ The precise coordination of these regulatory events is unknown, however through whole genome and exon sequencing, mutations within the Mediator complex have been implicated in developmental disorders.^6^ The Mediator complex is a dynamic structure responsible for integrating cellular signaling events with transcription by forming the preinitiation complex.^7^ This culminates in enhancer‐dependent regulation of RNA polymerase II, which is crucial for cellular regulation. The Mediator consists of a core 26 subunits in mammals^7^ and is divided into four submodules including the kinase submodule (CKM). The CKM is a reversibly dissociating submodule that consists of four proteins: MED13, MED12, CDK8, and CCNC in a stoichiometric ratio. In mammals, there are also paralogs for various CKM components, including MED13L for MED13, MED12L for MED12, and CDK19 for CDK8.^8^ The known function of each paralog in mammals in vivo is limited, but these paralogs are mutually exclusive within the Mediator.^9^

Mutations in *MED13*, *MED13L*, *CDK8*, *CDK19*, *MED12*, and *MED12L* have been reported to result in similar neurological disorders.^10^, ^11^, ^12^, ^13^, ^14^, ^15^, ^16^, ^17^ *MED13L* syndrome is generally characterized by a haploinsufficiency of MED13Lwith a broad phenotypic response. The most prevalent phenotypes, irrespective of mutation type, are intellectual disability (ID), developmental delay (DD), and facial dysmorphisms. Congenital heart defects have also been associated with a subset of individuals with *MED13L* syndrome. The syndrome is linked with a wide range of de novo mutations in *MED13L*.^18^, ^19^, ^20^, ^21^, ^22^ Thus, the causation between mutation and phenotypic response and severity (i.e., genotype–phenotype relationship) is challenging to decipher. In zebrafish, a knockdown of *med13b*, the zebrafish ortholog to *MED13L*, led to decreased migration of neural crest cells resulting in cartilage structure deformities, thus connecting neural crest cell migration with a phenotype similar to *MED13L* syndrome.^14^ By studying rare genetic disorders in animal models, we bridge the gap between genetic mutations and their physiological manifestations, paving the way for innovative treatments.^23^ In this paper, we classify a *Med13l* HET mouse model through the deletion of exon 11 as a proposed candidate for future *MED13L* syndrome studies.

## 2 RESULTS

### 2.1 Classification of a *Med13l* HET mouse

**FIGURE 1:**
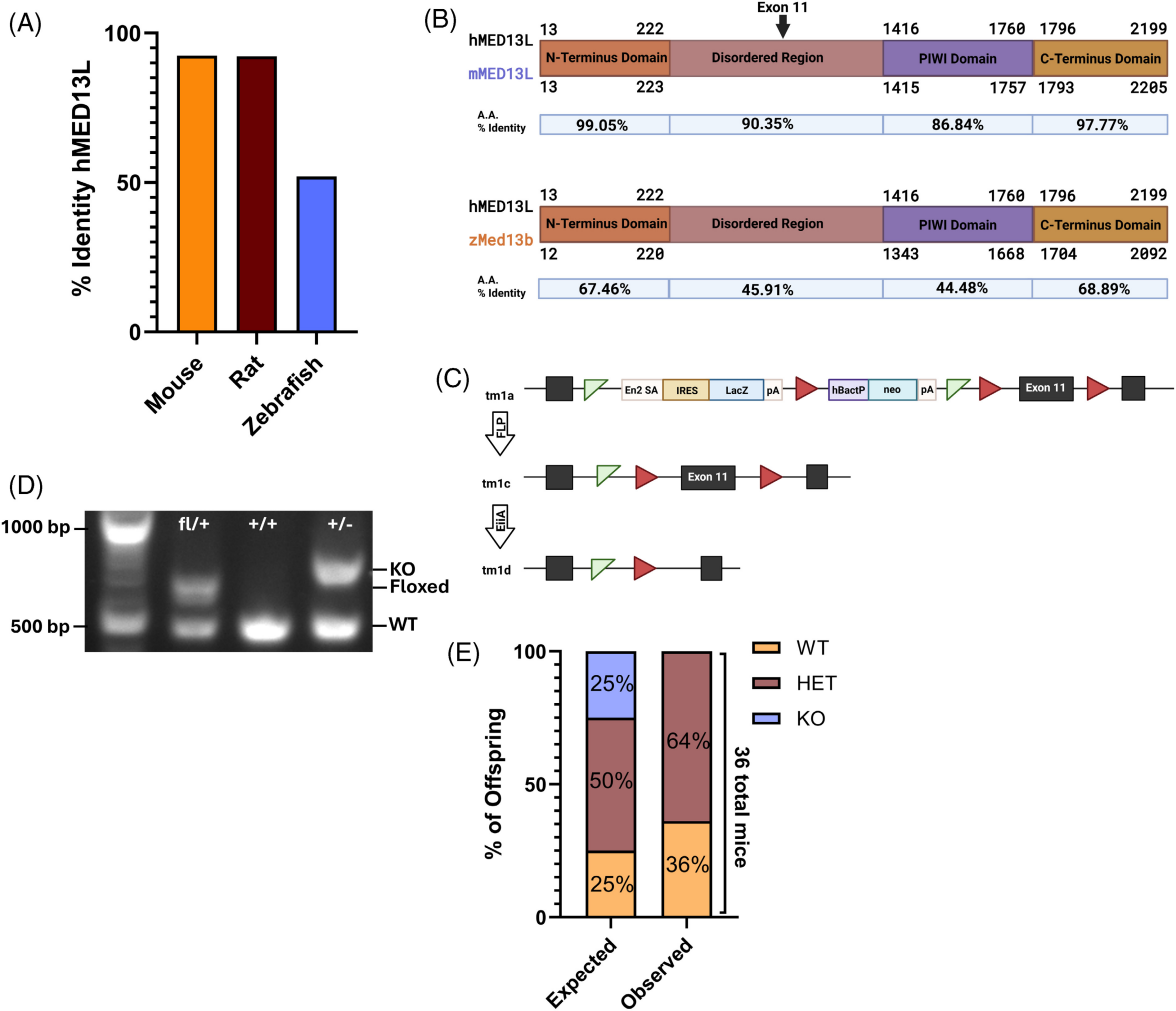
A *Med13l* HET mouse model generated based on sequence homology. (A) Percent similarity across species according to Clustal Omega alignment. (B) mMED13L and zMed13b percent similarity to hMED13L based on the N‐terminus, disordered region, PIWI, and C‐terminus domains. (C) A schematic for the KOMP KO 1st method used to remove exon 11 which consists of the 665th–741st amino acids in the disordered region. (D) Genotyping results identifying floxed (fl) (669 bp), knockout (KO) (686 bp), and wildtype (WT) (485 bp) alleles. (E) A mouse offspring chart (*n* = 36).

Previous studies modeled various phenotypic aspects of *MED13L* syndrome via knockdown approaches in zebrafish.^14^ However, only 52.60% of Med13b aligns with human MED13L whereas MED13L in mouse and rat align with 92.46% and 92.19% of the human gene (Figure 1A). To generate our *Med13l* HET mouse, we created a germline deletion of exon 11 which consist of the 665th–741st amino acids in the disordered region. The disordered region in mMED13L aligns with 90.35% of the disordered region in hMED13L (Figure 1B). We used the Komp KO 1st method (Figure 1C) and confirmed by PCR (Figure 1D). Due to embryonic lethality, we characterize the *Med13l* heterozygous germline deletion (Figure 1E).

### 2.2 *Med13l* HET mice display growth abnormalities

**FIGURE 2:**
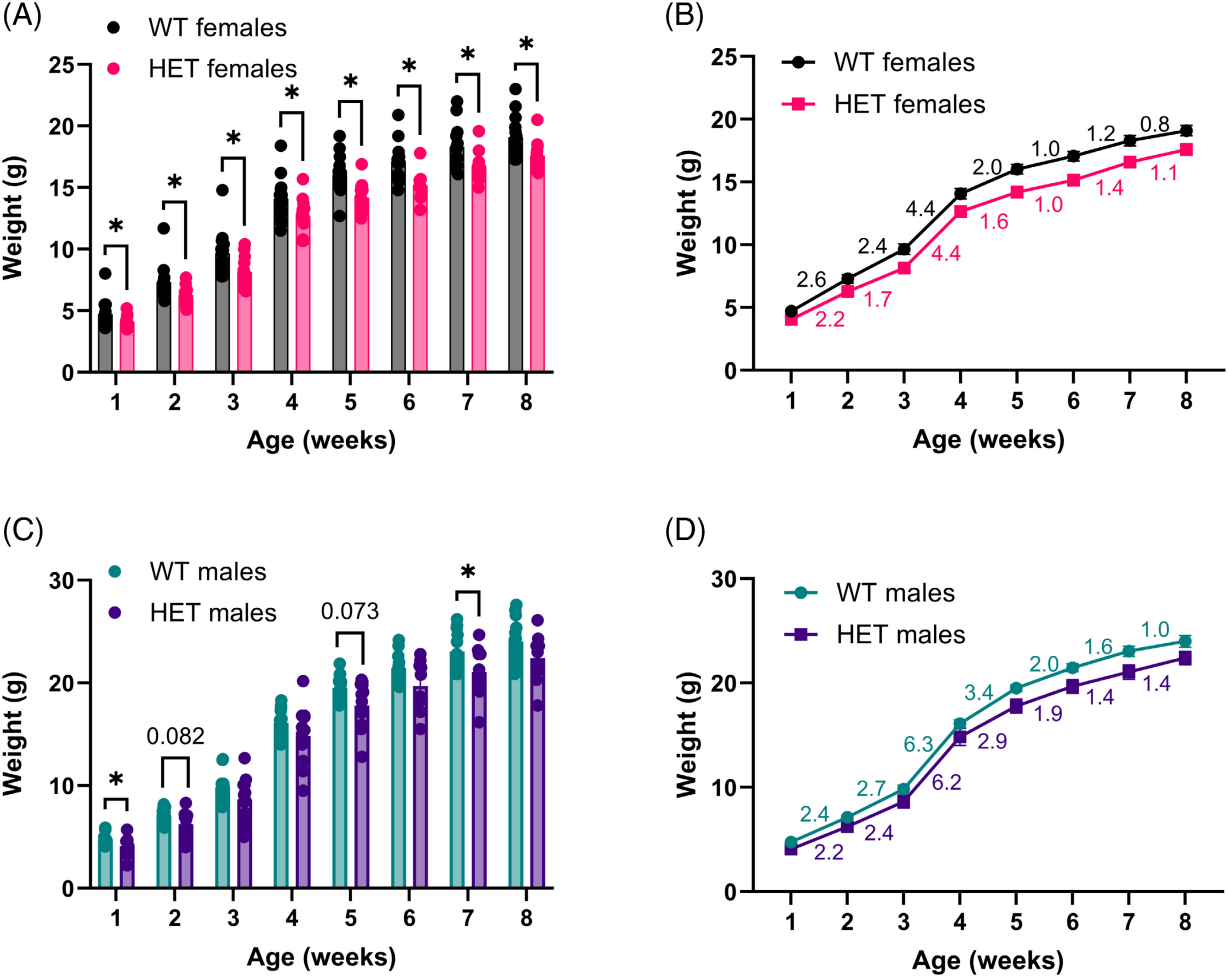
A growth analysis of WT and *Med13l* HET mice. (A) Weights for *Med13l* HET (*n* = 13) and WT (*n* = 16) females from P7 to 8 weeks of age. (B) Growth curves for *Med13l* HET and WT females and the rate of change between the average weight each week. (C) Weights for *Med13l* HET (*n* = 12) and WT (*n* = 14) males from P7 to 8 weeks of age. (D) Growth curves for *Med13l* HET and WT males and the rate of change between the average weight each week.

*MED13L* syndrome patients, irrespective of mutation type, have some form of developmental delay.^18^, ^19^, ^20^, ^21^, ^22^ The *Med13l* HET mice also showcase a developmental delay in terms of growth. The female HETs are significantly smaller than their WT counterparts over the course of 8 weeks (Figure 2A), but they follow the same growth curve and rate of growth as the WTs with the largest jump in growth occurring between week 3 and week 4 (Figure 2B). In terms of the males, the *Med13l* HET mice are significantly smaller than their WT counterparts at P7 and 7 weeks of age (Figure 2C). Like the females, the male *Med13l* HETs follow the same growth pattern as the WT males and experience similar growth rates compared to the WTs (Figure 2D). Thus, for both sexes, the *Med13l* HETs appear to start out smaller and never make up for the initial difference seen at P7.

### 2.3 Cardiac function for *Med13l* HET mice

**FIGURE 3:**
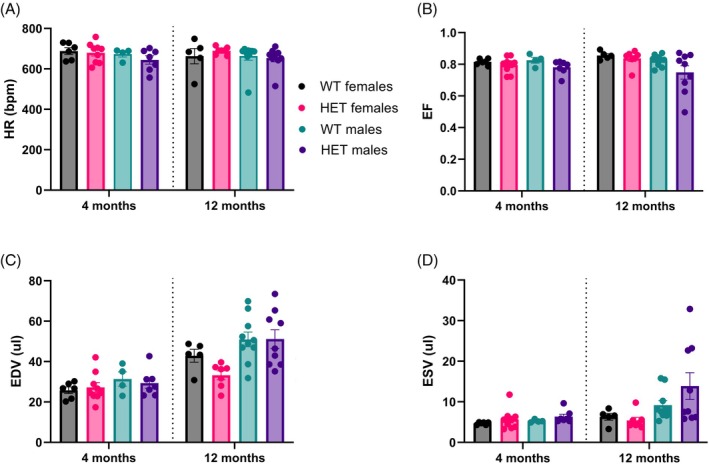
*Med13l* HET mice do not exhibit heart defects at 4 months or at a year‐old. Echocardiogram results from *Med13l* HET and WT mice at 4 months and a year old for (A) heart rate (HR), (B) ejection fraction (EF), (C) end‐diastolic volume (EDV), and (D) end‐systolic volume (ESV). At 4 months, the female cohort consists of *n* = 9 *Med13l* HETs and *n* = 6 WTs, and the male cohort consists of *n* = 7 *Med13l* HETs and *n* = 4 WTs. At a year old, the female cohort consists of *n* = 7 *Med13l* HETs and *n* = 5 WTs, and the male cohort consists of *n* = 9 *Med13l* HETs and *n* = 10 WTs. The 1‐year‐old mice were part of the 4‐month‐old cohort.

Congenital heart defects are present in 20%–50% of patients with *MED13L* syndrome.^19^ Thus, we conducted echocardiograms on the WT and *Med13l* HET cohorts at 4 months of age to determine whether the *Med13l* HETs showed any cardiac decline. The *Med13l* HET mice did not exhibit any differences in heart rate (HR), ejection fraction (EF), end‐diastolic volume (EDV), or end‐systolic volume (ESV) at 4 months of age (Figure 3A–D). Therefore, the deletion of *Med13l* exon 11 in mice does not induce structural or functional heart defects.

Little is known about age induced cardiac decline in *MED13L* syndrome, so we investigated if our HET mice experience cardiac decline due to aging alone. Both WT and *Med13l* HET mice underwent echocardiograms at 12 months of age. This cohort was made up of the same mice that underwent echocardiograms at 4 months. We did not observe any statistically significant differences when comparing WT and *Med13l* HET mice within the female and male cohorts (Figure 3).

### 2.4 Craniofacial anomalies in *Med13l* HET mice

**FIGURE 4:**
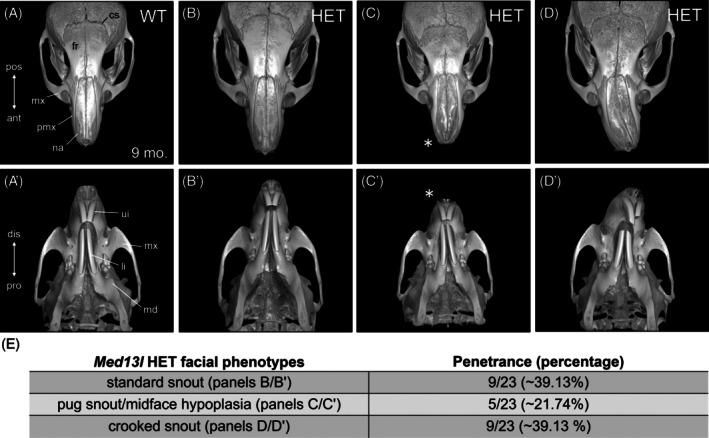
*Med13l* HET mice exhibit midfacial anomalies. Representative micro‐computed tomography surface renderings of 9‐month‐old female WT (*n* = 3) and *Med13l* HET mice (*n* = 6). Surface renderings are viewed in (A–D) top‐down and (A′–D′) bottom‐up fashions. Panels (B) and (B′) show a representative HET with relatively normal facial phenotypes; Panels (C) and (C′) show a representative HET with a pug snout/midface hypoplasia (asterisk); and Panels (D) and (D′) show a representative HET with a crooked snout and malocclusion. (E) Penetrance of facial anomalies in HET conditions, which includes both males and females (*n* = 23). ante, anterior; cs, coronal suture; dis, distal; fr, frontal bone; li, lower incisor; md, mandible; mx, maxilla; na, nasal bone; pmx, premaxilla; pos, posterior; pro, proximal; ui, upper incisor.

**FIGURE 5:**
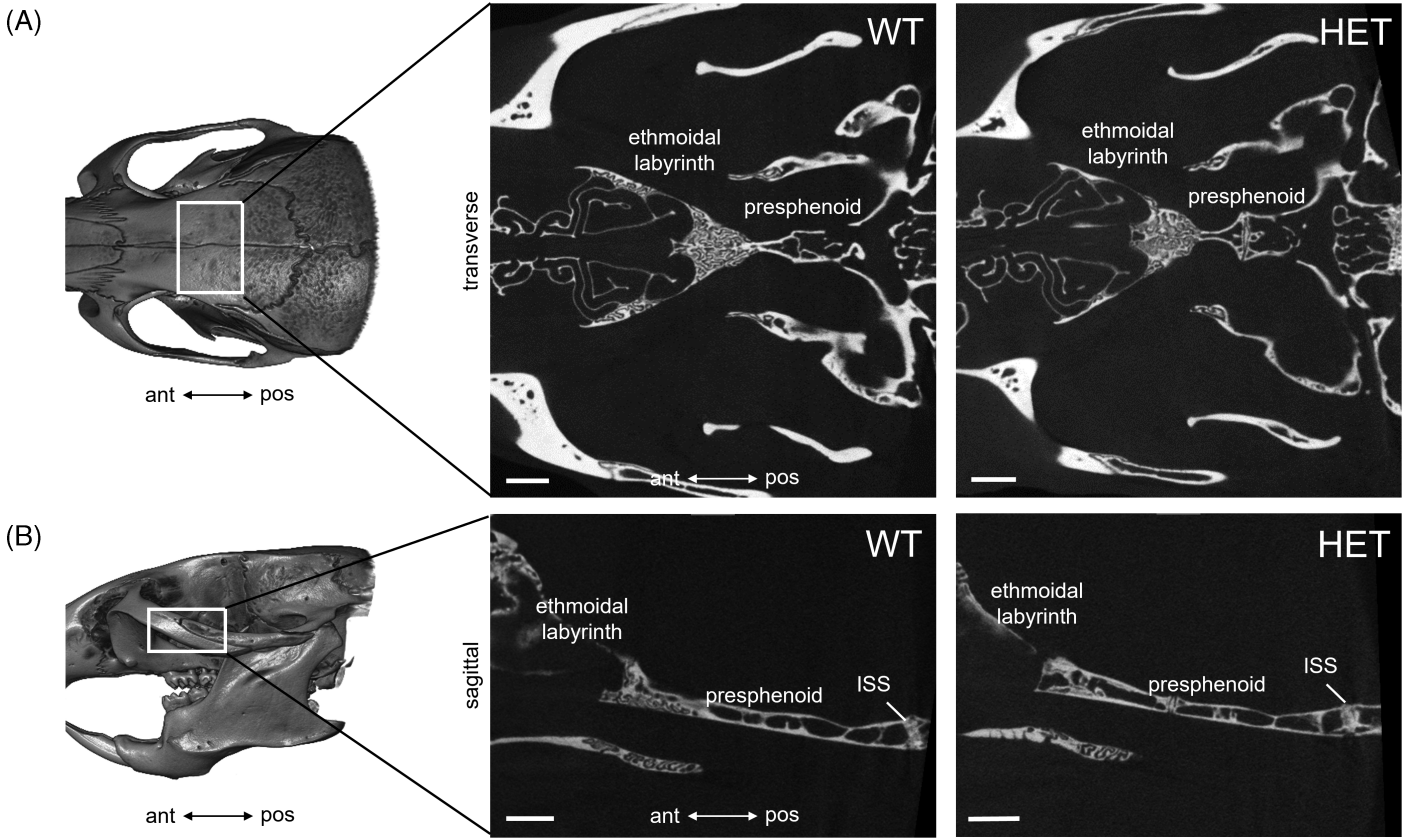
*Med13l* HET mice display normal anterior cranial base formation. (A, B) Micro‐computed tomography slices (A) transverse and (B) sagittal slices of the anterior cranial base and ethmoidal labyrinth are displayed, with indicated genotypes. Note, *Med13l* HET mice display normal anterior cranial base formation. ante, anterior; ISS, intersphenoid synchondrosis; post, posterior. Scale bar: 1 mm.

**FIGURE 6:**
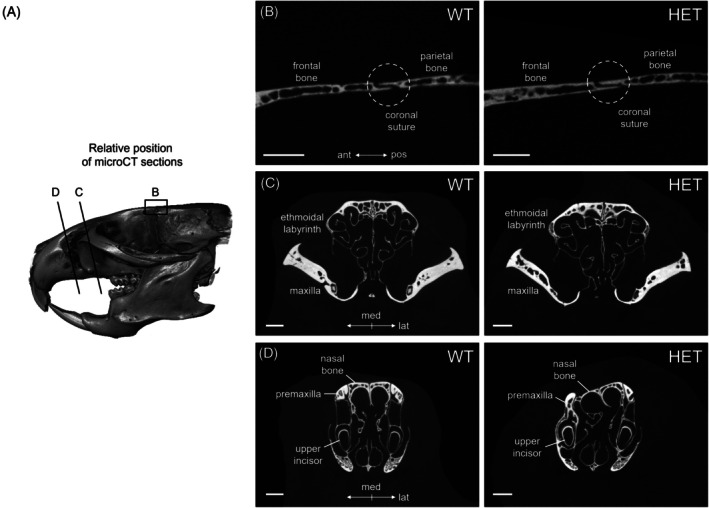
*Med13l* HET mice do not display patency defects in the nasal bones and coronal suture. (A) Representative micro‐computed tomography surface rendering of the adult mouse head, showing relative position and orientation of tomography slices for the subsequent figure panels. (B) Sagittal slices of the coronal suture (circled), (C) frontal slices of the ethmoidal labyrinth, and (D) frontal slices of the nasal skeletal elements. Note, no patency defects in the nasal bones and coronal sutures are observed in *Med13l* HET mice. ant, anterior; lat, lateral; med, medial; pos, posterior. Scale bar: 1 mm.

**FIGURE 7:**
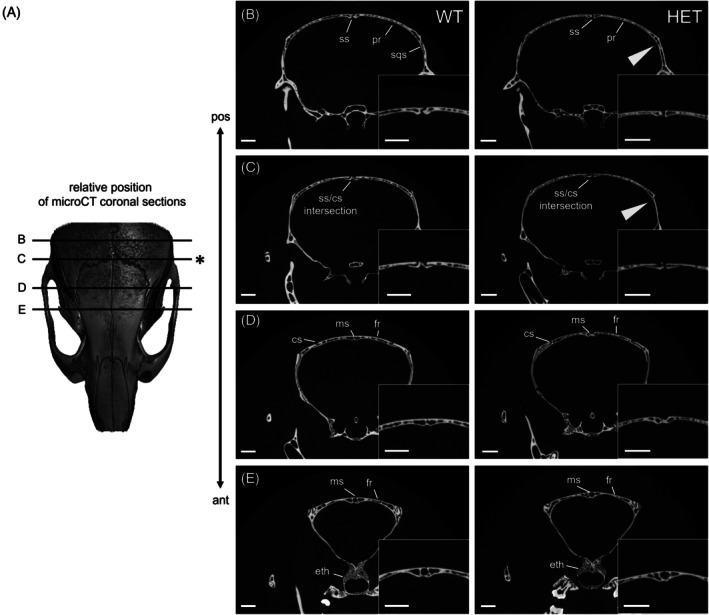
MicroCT slices show unilateral discontinuations of the squamosal suture in *Med13l* HET mice. (A) Representative micro‐computed tomography surface rendering of the adult mouse head, showing relative positions of serial, frontal slices through the calvaria (posterior to anterior). (B) Posterior to the coronal suture, displaying the sagittal suture (ss), parietal bones (pr), and squamosal suture (sqs). (C) Intersection of the sagittal suture and coronal suture (cs). (D) Anterior to the sagittal/coronal suture intersection, displaying the lateral regions of the coronal suture, the metopic suture (ms), and frontal bones (fr). (E) Anterior to panel (D), as indicated by the posterior regions of the ethmoidal labyrinth (eth). Inset is a magnified view of the midline suture displayed in each respective figure panel. Arrowhead points to the unilateral absence of the squamosal suture. ant, anterior; pos, posterior. Scale bar: 1 mm.

**FIGURE 8:**
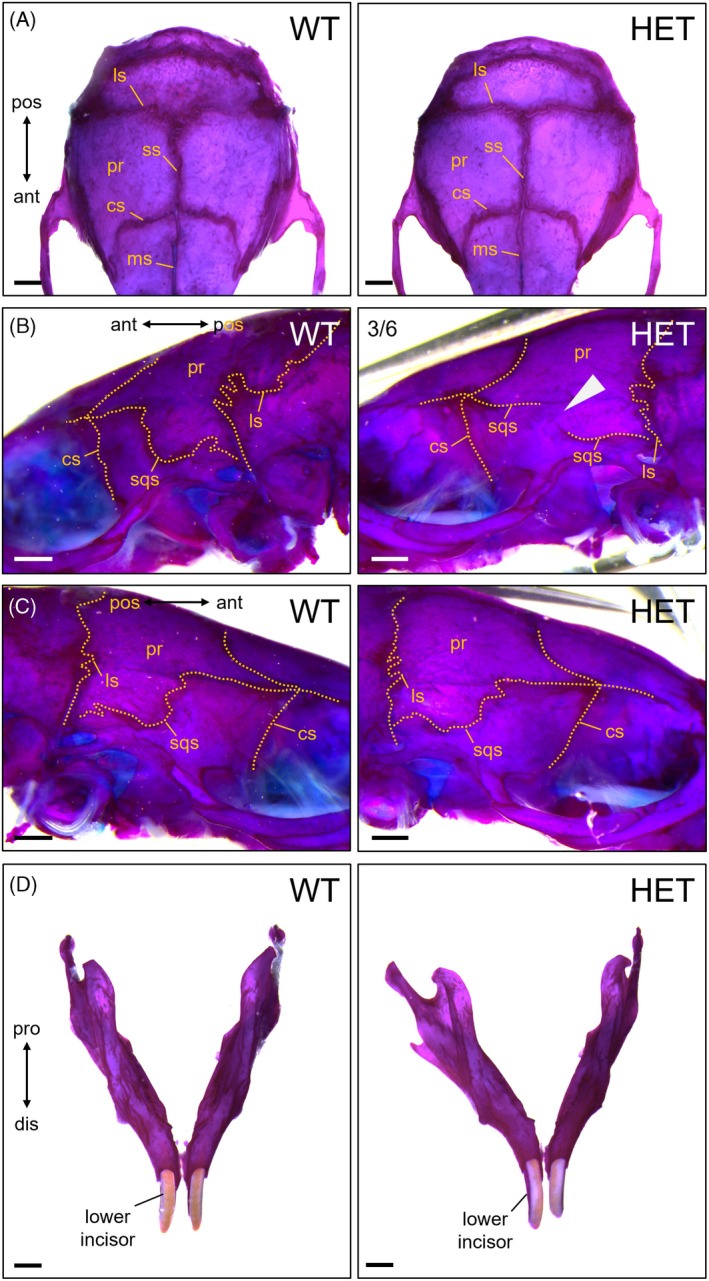
Skeletal staining further confirms *Med13l* HET mice display unilateral discontinuations of the squamosal suture. (A–D) Representative skeletal staining preparations of female WT and *Med13l* HET mice. (A) Top‐down view of the posterior calvaria, as indicated by the lambdoid suture (ls), the sagittal suture (ss), coronal sutures (cs), the metopic suture (ms), and parietal bones (pr). (B) Left and (C) right lateral views of the calvaria. Outlined in gold are the sutures. Arrowhead indicates discontinuation of the squamosal suture (sqs) (occurring in three of six HET mice irrespective of laterality and the presence of the crooked snout). (D) Mandibles in isolation viewed ventrally, with lower incisors pointing down. ante, anterior; d, distal; post, posterior; pro, proximal. Scale bar: 1 mm.

Along with developmental delay and congenital heart defects, *MED13L* syndrome patients also have craniofacial dysmorphisms such as a bulbous nose, flat nasal root, and bitemporal narrowing.^18^, ^19^, ^20^, ^21^, ^22^ At birth, *Med13l* HET mice look indistinguishable from their WT littermates. Interestingly, craniofacial phenotypes appear postnatally. While ~39% of *Med13l* HET mice display comparable craniofacial skeletons compared to WT mice at 9 months of age, the remaining ~61% display anomalies of the midfacial elements as shown by micro‐computed tomography (microCT) surface renderings (Figure 4A–E,A′–D′). Specifically, ~22% of *Med13l* HET mice exhibit a pug snout with midface hypoplasia (Figure 4C,C′, asterisk), while ~39% display a crooked snout (Figure 4D) that results in malocclusion (Figure 4D′) depending on the severity of the crooked snout. Fusion of various skeletal elements through the head, such as craniosynostosis are one possible source of improper craniofacial outgrowth. As such, we queried various craniofacial elements in the microCT‐scanned heads to identify potential differences between genotypes. Examining the ethmoidal labyrinth and anterior elements of the cranial base, *Med13l* HET mice display subtle differences in morphologies, however no obvious bone fusion defects were observed compared to WT mice (Figure 5A,B). Similarly, as in WT mice, the coronal sutures and nasal bone elements are not fused, even within the crooked snout of *Med13l* HET mice (Figure 6A–D). Interestingly, a subset of *Med13l* HET mice (three of six heads scanned) display unilateral discontinuations in the squamosal suture across select microCT slices (Figure 7A–E, arrowheads), irrespective of laterality and the presence of the crooked snout. We stained the microCT‐scanned heads with Alizarin red and Alcian blue as an orthogonal approach and confirmed the presence of the unilateral discontinuations along the squamosal suture (Figure 8A–C, arrowhead). Finally, consistent with the crooked snout being the major contributor to malocclusion, *Med13l* HET mice do not exhibit defects in the mandible compared to WT controls when examined in isolation (Figure 8D).

As shown previously by lineage tracing,^14^, ^26^, ^27^ the craniofacial elements affected in *Med13l* HET mice are derived from neural crest cells. However, such studies observed defective cranial development at embryonic/larval stages, whereas, interestingly, neural crest‐derived craniofacial structures in *Med13l* HET mice grow abnormally as they age after birth. Altogether, *Med13l* HET mice grow abnormally as they age birth. Altogether, *Med13l* HET mice recapitulate the craniofacial differences observed in *MED13L* syndrome patients.

## 3 CONCLUSIONS AND DISCUSSION

*MED13L* syndrome is a rare haploinsufficiency caused by de novo mutations in the *MED13L* gene. MED13L is part of the CKM which is a subunit of the Mediator complex. The Mediator complex is a dynamic structure that communicates regulatory signals between transcription factors and RNA Pol II which is paramount for gene regulation. *MED13L* syndrome is characterized by a broad phenotypic response and is caused by a wide range of de novo mutations.^18^, ^19^, ^20^, ^21^, ^22^ Hence, the correlation between mutation and phenotypic outcome is challenging to delineate. We classify a *Med13l* HET mouse model as a possible resource to begin deciphering the correlation between mutations and phenotypes.

The human and mouse MED13L proteins have significant overlap (Figure 1) making our *Med13l* HET mouse model a great choice to investigate *MED13L* syndrome. Complete loss of *Med13l* in mice is embryonic lethal, consistent with previous reports of MED13L's role in the 8cell‐to‐morula stage and necessity in preimplantation.^28^ While *Med13l* HETs are viable, males and females showcase a physical developmental delay which is more prominent in females. We believe this prominence is due to inherent variability and less of a gender specificity. Even though *Med13l* HET mice are smaller, they follow similar growth curves and experience similar growth rates to their WT counterparts (Figure 2). This suggests their differences in size are due to the *Med13l* HETs' inability to make up for the initial size difference observed at P7. Developmental delays are one of the more prevalent phenotypes seen in *MED13L* syndrome.^18^, ^19^, ^20^, ^21^, ^22^ Congenital heart defects, although less prevalent, are another phenotype seen in *MED13L* syndrome, yet our *Med13l* HET mice do not exhibit any structural or functional cardiac defects at 4 months of age (Figure 3). Future investigations should explore whether other mutations in *Med13l* cause functional and structural cardiac defects in mice.

Previous zebrafish models have shown a knockdown of *med13b* leads to defective neural crest cell migration resulting in stunted midfacial growth at 5 days post fertilization (dpf).^14^ Our *Med13l* HET mice also exhibit craniofacial anomalies in neural crest derived regions along with discontinuous squamosal sutures (Figures 4 and 5). Three de novo missense *MED13L* mutations have been associated with sagittal and metopic craniosynostosis.^29^ The sagittal, metopic, and squamosal suture are all derived from neural crest cell populations.^27^ Previous work has shown a slight increase in the proliferative capacity of knockdown *Med13l* neural progenitor cells.^14^ Therefore, the midfacial anomalies we observe along with the squamosal suture discontinuities could stem from either defective neural crest cell migration or a dysregulation in cell proliferation. This implies MED13L may function in neural crest cells to regulate craniofacial growth and differentiation. Future studies should focus on delineating the role of MED13L in neural crest cell regulation.

Interestingly, the midfacial anomalies we observe (e.g., a pug or crooked snout) in *Med13l* HETs are not visible until approximately weaning age, perhaps due to a compensatory mechanism initiated by MED13L's paralog, MED13. Previous work has shown MED13L can partially compensate for the loss of MED13 in preimplantation embryos and basal cardiac function in mice.^28^, ^30^ Redundancies have also been observed between other Mediator subunits such as CDK8 and CDK19 in intestinal cells.^31^, ^32^ Along with further elucidation of the compensatory mechanisms between MED13L and MED13, future research should investigate the interactions between MED13L and other Mediator subunits like CCNC. Along with protein interactors, identifying the cellular and molecular underpinnings of pathology in *MED13L* syndrome will be critical. Recent studies using *MED13L* syndrome human fibroblasts have identified abnormal CCNC nuclear release inducing mitochondrial fragmentation and dysfunction.^33^ Thus, several aspects of MED13L function and the association of these functions with *MED13*L syndrome pathology remain to be resolved. Importantly, our study has generated a novel mouse model that recapitulates *MED13L* syndrome phenotypes and will be a valuable tool for future studies in deciphering molecular and pathophysiological mechanisms driving *MED13L* syndrome.

## 4 EXPERIMENTAL PROCEDURES

### 4.1 Generation of a *Med13l* HET mouse

The mouse strain used for this project, C57BL/6N‐*Med13ltm1a(KOMP)Wtsi*/MbpMmucd, RRID:MMRRC_048567‐UCD, was obtained from the Mutant Mouse Resource and Research Center (MMRRC) at the University of California, Davis, an NIH‐funded strain repository. The strain was donated to the MMRRC by The KOMP Repository, University of California, Davis; Originating from Kent Lloyd, UC Davis Mouse Biology Program. The KOMP Tm1d KO‐first method was followed to generate the *Med13l* HET mouse. Briefly, a mouse harboring the Tm1a tKO first *Med13l* allele was bred to mice containing the Flpe recombinase allele followed by resulting offspring being bred to mice containing the Eiia cre recombinase allele, generating a null (i.e., tm1d) *Med13l* allele in the final offspring. Both Flpe and Eiia cre alleles were removed by out‐crossing. Genotyping was performed with a single forward primer: 5′‐CGG GGA GTG TCT CAA AAT CAA ACC C‐3′ and one of two reverse primers: 5′‐CCA AGC CTC TGG ATC TAC ATA GAC CC‐3′ to distinguish the knockout (KO) allele, and 5′‐CCT TCT CCC CAG TCC AGT AGT TAC C‐3′ to distinguish the wildtype (WT) and the floxed (fl) allele. The expected band sizes are 686 base pairs (bp) for the KO allele, 485 bp for the WT allele, and 669 bp for the fl allele. After generation of *Med13l* HET mice, all breeding schemes were a *Med13l* HET male to a C57BL/6 NJ female. All mice were used in accordance with policies approved by the Institutional Animal Care and Use Committee at the University of Iowa. All mice were fed a standard chow diet and given water and food ad‐libitum. All mice were housed in AAALAC housing on a 12/12‐h light/dark cycle.

### 4.2 Clustal Omega analyses

FASTA protein sequences were obtained from NCBI as follows: *Homo sapiens* MED13L (NP_056150.1), *Mus musculus* MED13L (NP_001334374.1), *Rattus norvegicus* Med13L (XP_038946101.1), and *Danio rerio* Med13b (NP_001077307.2). Domains were identified as follows: N‐terminal (CDD:463303), PIWI (CDD:465699), C‐terminal (CDD:461879). Clustal Omega was run using clustalo (1.2.4) with default Clustal Omega parameters: output guide tree (yes), distance matrix (no), dealing input (no), MBED‐like clustering guide tree (yes), MBED‐like clustering iteration (yes), combined iterations (default[0]), max guide tree (default), max hmm iterations (default), order (aligned). The output format was ClustalW.^24^

### 4.3 Statistics

Statistics were done in GraphPad Prism version 10.4.1. A nonparametric Mann–Whitney *U* test was used to compare the group medians between WT and *Med13l* HET mice within male and female cohorts. A *p*‐value <.05 was considered significant. When necessary, a Sidalk‐Holm multiple comparisons correction was used to compute adjusted *p*‐values. In terms of the echocardiogram data, statistical analyses were conducted comparing age and sex matched mice and were not conducted across time points or across sexes.

### 4.4 Mouse growth analyses

WT and *Med13l* HET male and female mice were weighed every 7 days starting at P7 until 8 weeks of age. Weights obtained from mice with malocclusions were not included in the growth analysis. Growth curves were created by plotting the average weight per group per week and computing the slope of the line between each average value.

### 4.5 Echocardiography

Cardiac function of both male and female WT and *Med13l* HET mice was assessed at 4 and 12 months of age. Cardiac function was measured via high‐frequency echocardiography (30 MHz) linear array transducer (Vevo 2100; Visual Sonics, Toronto, ON, Canada). Mice were not sedated as parasternal long and short axis views were obtained. Measurements were performed by a single experienced and blinded technician from the University of Iowa Cardiovascular Phenotyping Core.

### 4.6 Micro‐computed tomography analyses

Micro‐computed tomography (microCT) scanning was serviced through the University of Iowa Small Animal Imaging Core Facility. For sample preparation, 9‐month‐old mouse heads were first deskinned, fixed in 4% PFA for 3 days, and then washed in 1X PBS. The back of the heads was embedded in 1% low‐melt agarose (Research Products International, Mt. Prospect, IL), snout pointing vertically upward, in 50 mL conical tubes. Heads were scanned on a Zeiss Xradia 520 Versa system at pixel values of 15.5–17.2, with the field of vision displaying anterior portions of the heads (from the parietal bone forward). DICOM formats of the microCT scans were visualized on the Dragonfly software (Object Research Systems Inc., Montreal, Quebec), either as three‐dimensional surface renderings or optical slices.

### 4.7 Skeletal staining analyses

Nine‐month‐old mouse heads used in microCT analysis were subject to whole‐mount Alizarin Red/Alcian Blue staining,^25^ with modifications to soft tissue dissociation steps using potassium hydroxide (KOH) to account for extensive tissue fixation. Heads were incubated in acetone for 2 days at room temperature. Heads were then incubated in Alcian blue staining solution, comprised of 0.03% (w/v) Alcian blue 8GX powder (Sigma‐Aldrich) dissolved in 80% ethanol/20% glacial acetic acid. Excess staining solution was washed out in 95% ethanol overnight. Next, heads incubated in Alizarin red staining solution comprised of 0.005% (w/v) Alizarin red powder (Sigma‐Aldrich) dissolved in 1% KOH.

Finally, tongue and muscle tissue were removed using forceps, and heads rocked up to 3 weeks in KOH (replaced every 2–3 days) to remove remaining soft tissue. Specifically, heads first rocked in 2% KOH for 2 weeks; then, 6% KOH until majority of soft tissue was dissociated (2 days); finally, 2% KOH (2–3 days). In 20% glycerol, skeletal elements were stored at 4°C until imaged, in situ or in isolation, on a brightfield microscope paired with the LAS X imaging software (Leica Microsystems, Wetzlar, Germany).

## FUNDING INFORMATION

This work was funded by the NIH/NHLBI 1R01HL168044 (CEG), NIH Postdoctoral Training Grant T32DK112751 (AKL), NIH Predoctoral Training Grant T32GM145441 (KMH), the MED13L Foundation, NIH/NIDCR F31DE032881 (TTN), NIH/NIDCR R01DE033009 (EVO), and the University of Iowa College of Dentistry start‐up funds (EVO).

## CONFLICT OF INTEREST STATEMENT

The authors report no conflict of interest.

## Data Availability

All data is available in the manuscript or available by request from the corresponding author.
